# Oxidative and Endoplasmic Reticulum Stress Represent Novel Therapeutic Targets for Choroideremia

**DOI:** 10.3390/antiox12091694

**Published:** 2023-08-30

**Authors:** Hajrah Sarkar, Manuela Lahne, Neelima Nair, Mariya Moosajee

**Affiliations:** 1 UCL Institute of Ophthalmology, London EC1V 9EL, UK; 2 Francis Crick Institute, London NW1 1AT, UK

**Keywords:** choroideremia, oxidative stress, ER stress, neuroprotectants, zebrafish, patient fibroblasts

## Abstract

Choroideremia (CHM) is a rare X-linked chorioretinal dystrophy, affecting the photoreceptors, retinal pigment epithelium (RPE) and choroid, with no approved therapy. CHM is caused by mutations in the *CHM* gene, which encodes the ubiquitously expressed Rab escort protein 1 (REP1). REP1 is involved in prenylation, a post-translational modification of Rab proteins, and plays an essential role in intracellular trafficking. In this study, we examined oxidative and endoplasmic reticulum (ER) stress pathways in *chm^ru848^* zebrafish and *CHM^Y42X^* patient fibroblasts, and screened a number of neuroprotectants for their ability to reduce stress. The expression of the oxidative stress markers *txn*, *cat* and *sod3a*, and the ER stress markers *bip*, *atf4* and *atf6*, were dysregulated in *chm^ru848^* fish. The expression of *SOD2* was also reduced in *CHM^Y42X^* fibroblasts, along with reduced *BIP* and increased *CHOP* expression. The lack of REP1 is associated with defects in vesicular trafficking, photoreceptor outer segment phagocytosis and melanosome transport, leading to increased levels of stress within the retina and RPE. Drugs targeting oxidative and ER stress pathways represent novel therapeutic avenues.

## 1. Introduction

Choroideremia (CHM) is an X-linked chorioretinal dystrophy, caused by mutations in the *CHM* gene, encoding the ubiquitously expressed Rab escort protein 1 (REP1). CHM is a degenerative condition affecting the photoreceptors, retinal pigment epithelium (RPE) and choroid. Male patients typically present with nyctalopia in early childhood, progressing to peripheral visual field loss, and finally progressing to the loss of central vision and complete blindness by the fifth to sixth decade of life [1].

REP1 is involved in the prenylation, a post-translational lipid modification, of Rab proteins, which is essential for intracellular transport. REP1 acts as a chaperone by binding to unprenylated Rab proteins and transferring them to geranylgeranyl transferse II (GGTaseII) for the addition of a geranylgeranyl group to the C-terminus of the protein; it then transports the modified Rab proteins to target intracellular membranes [2]. The loss of REP1 results in an accumulation of unprenylated Rabs and has been linked to defects in vesicular trafficking, phagocytosis and melanosome transport [3,4,5,6]. In most tissues except the retina, REP2, encoded by the CHM-like (*CHML*) gene, compensates for the lack of REP1, restricting pathogenesis to the eye. However, we recently carried out a whole-metabolomic screening of 25 patients and 25 age-matched controls and reported a range of altered metabolites in the CHM patient plasma, including those associated with oxidative stress; this indicates possible systemic manifestations, which may also be reflective of retinal status [7].

There are currently no approved therapies for CHM, although several clinical trials for adeno-associated viral (AAV) gene therapy have taken place. Despite promising early results [8,9], a recent phase 3 clinical trial for timrepigene emparvovec (BIIB111/AAV2-REP1) reported a failure to meet primary and secondary endpoints; therefore, an investigation of alternative therapies is required. Given the recent reports of possible systemic manifestations, the systemic application of neuroprotectants may be beneficial. In this paper, we characterise oxidative and endoplasmic reticulum (ER) stress in two models of CHM: (i) The *chm^ru848^* zebrafish which, due to the lack of a compensatory REP2 homolog, displays a multisystemic degenerative phenotype with small eyes, a shortened body length, oedema and an average survival of 4.8 days post fertilisation (dpf); by 5 dpf, there is widespread retinal cell death with a loss of lamination and the RPE is hypertrophic [10]. (ii) Dermal fibroblasts from a 28-year-old male CHM patient with the c.126C>G; p.Tyr42* variant, which has no detectable REP1 protein expression or prenylation activity [11]. In addition, we test a panel of drugs that have shown positive indications in other models of retinal disease and analyse them for their ability to reduce stress and improve the phenotype in CHM models.

## 2. Materials and Methods

### 2.1. Zebrafish Husbandry

Wild-type AB (wt) and choroideremia (*chm^ru848^*) zebrafish were bred and maintained according to local UCL and U.K. Home Office regulations for the care and use of laboratory animals under the Animals Scientific Procedures Act at the UCL Bloomsbury campus zebrafish facility. Zebrafish were raised at 28.5 °C on a 14 h light/10 h dark cycle. The UCL Animal Welfare and Ethical Review Body approved all procedures for experimental protocols, in addition to the U.K. Home Office (License no. PPL PC916FDE7). All approved standard protocols followed the guidelines of the ARVO Statement for the Use of Animals in Ophthalmic and Vision Research Ethics. Zebrafish were terminally anaesthetised in 0.2 mg/mL Tricaine (MS-222) for sample collection.

### 2.2. Drugs

N-acetylcysteine amide (NACA) was obtained from TOCRIS. Taurordeoxycholic acid (TUDCA) and L-dopa were purchased from Cayman Chemicals and taurine was purchased from Sigma (St. Louis, MO, USA). A stock solution of TUDCA was prepared in dimethylsulfoxide (DMSO). All other drug stocks were prepared in sterile water.

### 2.3. Zebrafish Dosing

Drugs were prepared in E3 medium and applied directly to embryos at either 10 hpf or 3 dpf, and collected at 5 dpf for further analysis. The wildtype fish were initially treated with a range of concentrations and the optimal dosing concentration was determined, based on 100% survival at 5 dpf with normal morphology. For each treatment, 50 embryos were used and three independent experiments were performed.

### 2.4. Fibroblast Cell Culture and Dosing

Human dermal fibroblasts from a patient with the *CHM* (NM_000390.4) variant c.126C>G; p.Tyr42* (*CHM^Y42X^*) and an age-matched control (WT) were generated, as previously described [11]. Cells were maintained in DMEM high glucose, 15% FBS and penicillin/streptomycin (Thermo Fisher Scientific, Waltham, MA, USA). To determine the optimum dosing concentrations, cells were treated with various concentrations of NACA and taurine for 24 h and the cell viability was assessed using MTT assay. TUDCA concentration was based on a previous publication [12]. For dosing experiments, cells were plated in 6-well plates at a seeding density of 600,000 cells per well. After 24 h, drugs were added to the cells in culture media. Twenty-four hours later, cells were pelleted and stored at −80 °C for further analysis.

### 2.5. RT-qPCR

Total RNA was extracted from cells using the RNeasy mini kit and from dissected eyes using the RNeasy FFPE kit (Qiagen, Hilden, Germany). cDNA was synthesised from 1 μg of RNA using the Superscript II First Strand cDNA synthesis kit (Thermo Fisher Scientific), according to the manufacturer’s instructions. Transcript levels were analysed using SYBR Green MasterMix (Thermo Fisher Scientific) on a StepOne Real-Time PCR system (Applied Biosystems, Thermo Fisher Scientific, Waltham, MA, USA), under standard cycling conditions. All samples were assayed in triplicate. The primer sequences are shown in Table 1.

### 2.6. Western Blot

Samples were analysed using western blot, as previously described [12], using anti-BIP (1:1000; Abcam ab21685, Cambridge, UK) overnight at 4 °C. The membrane was stripped and re-probed with anti-β-actin for 2 h at room temperature (1:5000; Sigma A2228).

### 2.7. SOD Activity Assay

SOD activity was determined using the SOD assay kit (Sigma; 19160) according to the manufacturer’s instructions and normalised to the total protein concentration.

### 2.8. Retinal Histology

Embryos at 5 dpf were fixed in 4% paraformaldehyde overnight at 4 °C and embedded using the JB-4 embedding kit (Polysciences Inc., Warrington, PA, USA). Sections were cut at a thickness of 7 µm, stained with 1% toluidine blue and imaged on an a Axioplan 2 microscope (ZEISS Microscopy, Jena, Germany).

### 2.9. Melanin Quantification

The melanin content was determined according to the protocol used by Agalou et al. [13]. Briefly, 10 embryos were sonicated in cold lysis buffer (20 mM of sodium phosphate (pH 6.8), 1% Triton X-100, 1 mM of EDTA, 1× Halt protease and phosphatase inhibitors cocktail). An aliquot of the lysate was reserved to determine the protein content using the Pierce BCA protein kit (Thermo Fisher Scientific). The lysate was centrifuged at 10,000× *g* for 10 min. The pellet was resuspended in 1 mL of 1 N NaOH/10% DMSO and incubated at 95 °C for 1 h. Absorbance was measured at 405 nm. Data were normalized to the total protein content.

### 2.10. Statistical Analysis

All statistical analyses were performed using GraphPad Prism 9 and data are expressed as mean ± SEM from 3 replicates. For all analyses, the Shapiro–Wilk normality test was initially carried out to determine whether the data were normally distributed, and the appropriate statistical test was chosen. For a comparison between the two groups, the data were analysed using the Student’s t-test. For grouped analyses, one-way ANOVA with Sidaks multiple comparison test was used. A *p* value of ≤0.05 was considered statistically significant.

## 3. Results

### 3.1. Increased ER and Oxidative Stress in chm^ru848^ Zebrafish and CHM^Y42X^ Patient Fibroblasts

**Figure 1:**
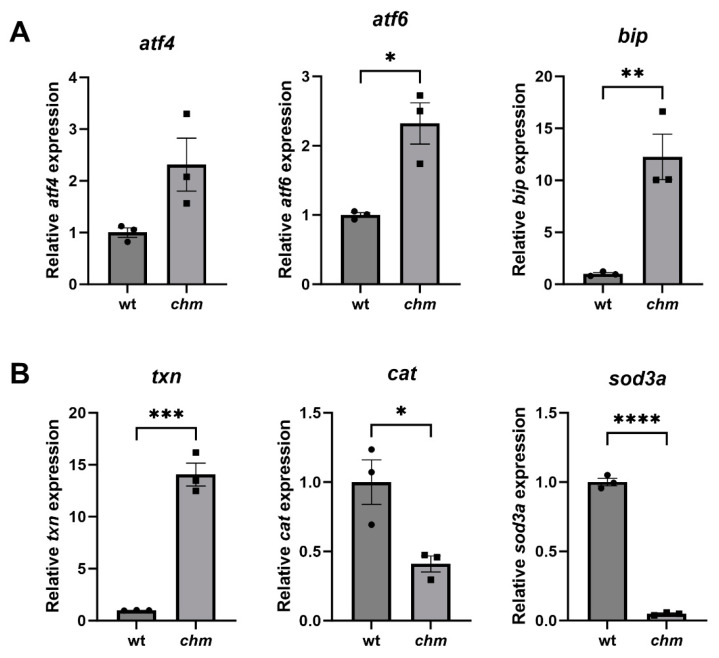
Increased ER and oxidative stress in *chm^ru848^* zebrafish. The expression of ER (**A**) and oxidative stress markers (**B**) were analysed in wt and *chm* fish at 5 dpf using RT-qPCR. ER and oxidative stress markers are differentially expressed in *chm* fish, compared to wt. Data expressed as mean ± SEM from *n* = 3 (denoted by black dots for wt and black squares for *chm* fish). * *p* ≤ 0.05, ** *p* ≤ 0.01, *** *p* ≤ 0.001, **** *p* ≤ 0.0001.

The expression of ER and oxidative stress markers was analysed using RT-qPCR in *chm^ru848^* zebrafish eyes at 5 dpf. The expression of the ER stress markers *atf6*, *atf4* and *bip* was significantly increased in *chm^ru848^* fish by 2.3 (*p* = 0.0116), 2.3 and 12.3-fold (*p* = 0.0068), respectively, compared to the wildtype (wt) fish (Figure 1A). The expression of the oxidative stress markers *cat* and *sod3a* was significantly reduced by 2.4— (*p* = 0.0258) and 20.7-fold (*p* < 0.0001), respectively, compared to wt fish, and the expression of *txn* was significantly increased by 14.1-fold (*p* = 0.0003) in *chm^ru848^* fish (Figure 1B).

**Figure 2:**
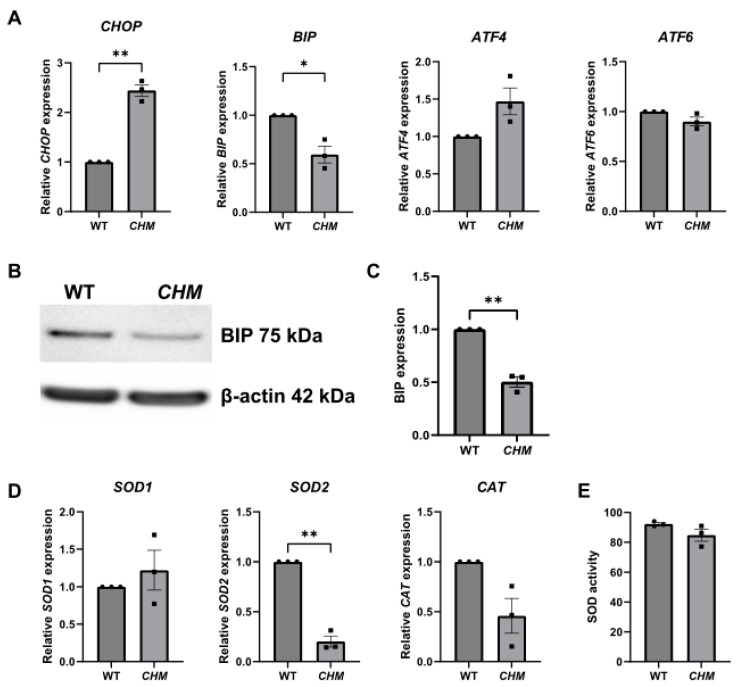
Increased ER and oxidative stress in *CHM^Y42X^* patient fibroblasts. (**A**) The expression of ER stress markers was analysed in WT and *CHM* fibroblasts using RT-qPCR. Data expressed as mean ± SEM from *n* = 3. (**B**) Representative western blot showing reduced BIP protein expression in *CHM* fibroblasts. (**C**) Quantification of BIP protein from *n* = 3 blots. (**D**) The expression of oxidative stress markers was analysed using RT-qPCR. (**E**) No significant difference in the SOD enzyme activity between WT and *CHM* fibroblasts. Data expressed as mean ± SEM from *n* = 3 (denoted by black dots for wt and black squares for *chm* fish). * *p* ≤ 0.05, ** *p* ≤ 0.01.

The expression of a number of ER- and oxidative-stress-related genes was analysed in *CHM^Y42X^* patient fibroblasts. The ER stress marker *BIP* was significantly decreased (*p* = 0.0421) and *CHOP* was significantly increased (*p* = 0.0068) in *CHM^Y42X^* patient fibroblasts compared to the WT cells (Figure 2A). Western blot analysis also revealed reduced BIP protein expression in *CHM^Y42X^* fibroblasts, compared to the WT (*p* = 0.0094) (Figure 2B,C). The only oxidative stress marker that was found to be significantly reduced by 5-fold (*p* = 0.0048) was *SOD2*, compared to the WT fibroblasts (Figure 2D). A SOD activity assay was carried out; however, there was no difference in the overall SOD activity between the WT and *CHM^Y42X^* fibroblasts (Figure 2E).

### 3.2. Drug Screening of Therapeutics Targeting ER and Oxidative Stress

A number of drugs that have previously been identified as having the ability to reduce ER and oxidative stress in other models of retinal disease were tested in the *chm^ru848^* zebrafish and *CHM^Y42X^* patient fibroblasts.

### 3.3. Tauroursodeoxycholic Acid (TUDCA)

**Figure 3:**
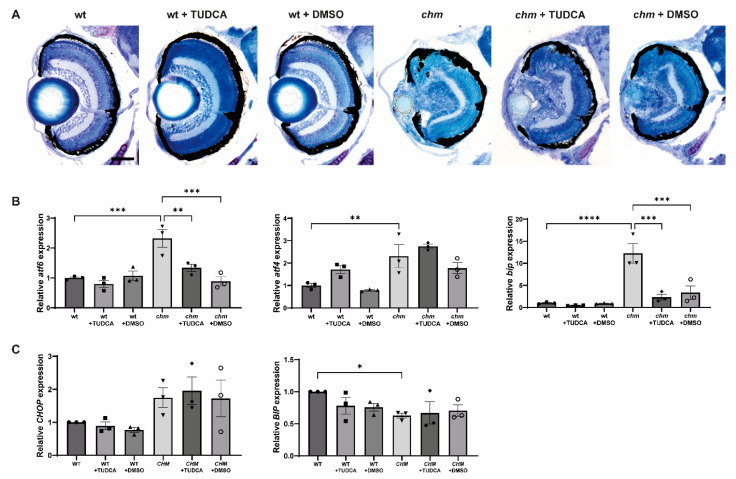
Analysis of ER stress in TUDCA-treated *chm^ru848^* fish and *CHM^Y42X^* cells. Fish were treated with 20 µM of TUDCA or DMSO at 3 dpf and collected at 5 dpf for (**A**) retinal histology and (**B**) the RT-qPCR analysis of the ER stress markers *atf6*, *bip* and *atf4*. TUDCA reduces the expression of ER stress markers. (**C**) Cells were treated with either 100 µM of TUDCA or DMSO and the expression of the ER stress markers *CHOP* and *BIP* was analysed using RT-qPCR. Data are expressed as mean ± SEM from *n* = 3. * *p* ≤ 0.05, ** *p* ≤ 0.01, *** < *p* ≤ 0.001 **** *p* ≤ 0.0001. Scale bar (50 μm), shown in wt corresponds to all images in panel.

TUDCA is a bile acid commonly used to treat liver conditions and has been shown to possess ER-stress-lowering properties. TUDCA has been reported to slow retinal degeneration in various mouse models of retinitis pigmentosa (RP) [14,15,16]. Zebrafish were treated with 20 µM TUDCA at 3 dpf and assessed at 5 dpf. Fish were also treated with an equivalent volume of DMSO as vehicle control. The retinal histology showed widespread retinal degeneration in *chm^ru848^* fish, including disrupted retinal lamination, areas of RPE atrophy and a small lens. Treatment with TUDCA did not result in any significant improvement in the retinal phenotype (Figure 3A). The ability of TUDCA to reduce the expression of ER stress markers in *chm^ru848^* fish was analysed using RT-qPCR, following TUDCA treatment. Although the expression of *atf6* (*p* = 0.0037) and *bip* (*p* = 0.0001) was significantly reduced in TUDCA-treated *chm^ru848^* fish, these markers were also significantly reduced in DMSO-treated *chm^ru848^* fish (*p* = 0.0002 and *p* = 0.0003, respectively), indicating that this effect is due to DMSO (Figure 3B). Next, we evaluated the ER-stress-lowering properties of TUDCA in *CHM^Y42X^* patient fibroblasts. Cells were treated with 100 µM of TUDCA or an equivalent volume of DMSO for 24 h, and the expression of *CHOP* and *BIP* was analysed using RT-qPCR; however, there was no significant reduction in ER stress in the TUDCA-treated cells (Figure 3C).

### 3.4. Taurine

**Figure 4:**
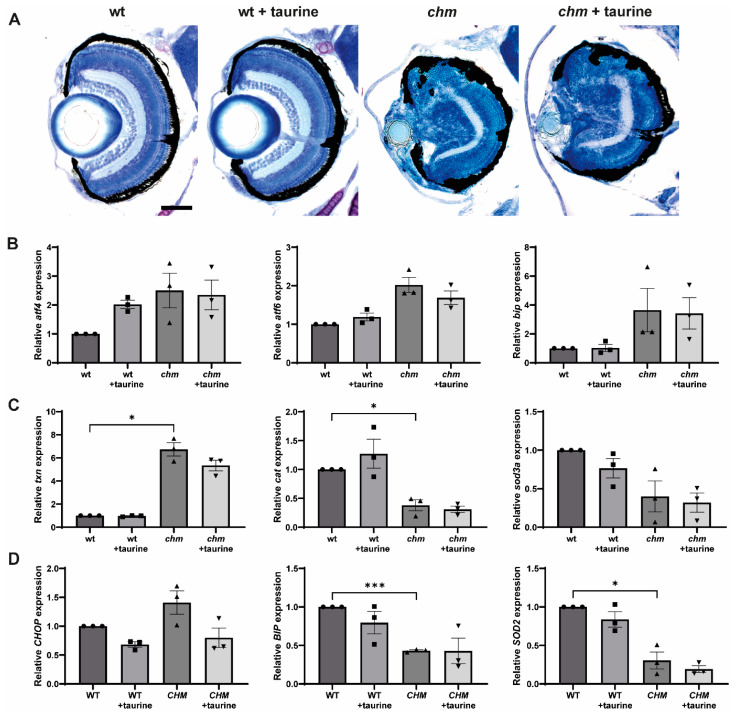
Analysis of ER stress in taurine-treated *chm^ru848^* fish and *CHM^Y42X^* cells. Fish were treated with 100 mM of taurine at 3 dpf and collected at 5 dpf for (**A**) retinal histology and RT-qPCR analysis of (**B**) the ER stress markers *atf6*, *bip* and *atf4* and (**C**) the oxidative stress markers *txn*, *cat* and *sod3a*. (**D**) Cells were treated with 100 mM of taurine and the expression of *SOD2* and ER stress markers *CHOP* and *BIP* was analysed using RT-qPCR. Data are expressed as mean ± SEM from *n* = 3. * *p* ≤ 0.05, *** < *p* ≤ 0.001. Scale bar (50 μm), shown in wt corresponds to all images in panel.

Taurine is a non-essential amino acid that is present at high concentrations in the retina, and has been shown to attenuate both ER and oxidative stress [17]. In one study, taurine was shown to rescue photoreceptor loss and visual function in an N-methyl-N-nitrosourea (MNU)-induced mouse model of photoreceptor degeneration [18]. Taurine was also shown to reduce starvation-induced ER stress in ARPE-19 cells [19]. Fish were treated with 100 mM of taurine at 3 dpf and the expression of ER and oxidative stress markers was analysed at 5 dpf. Taurine did not cause any significant reduction in the expression of ER or oxidative stress markers in *chm^ru848^* fish (Figure 4B,C). There was also no significant improvement in retinal histology (Figure 4A). *CHM^Y42X^* fibroblasts were treated with 100 of mM taurine for 24 h; however, there was no significant reduction in ER or oxidative stress (Figure 4D).

### 3.5. N-Acetylcysteine Amide (NACA)

**Figure 5:**
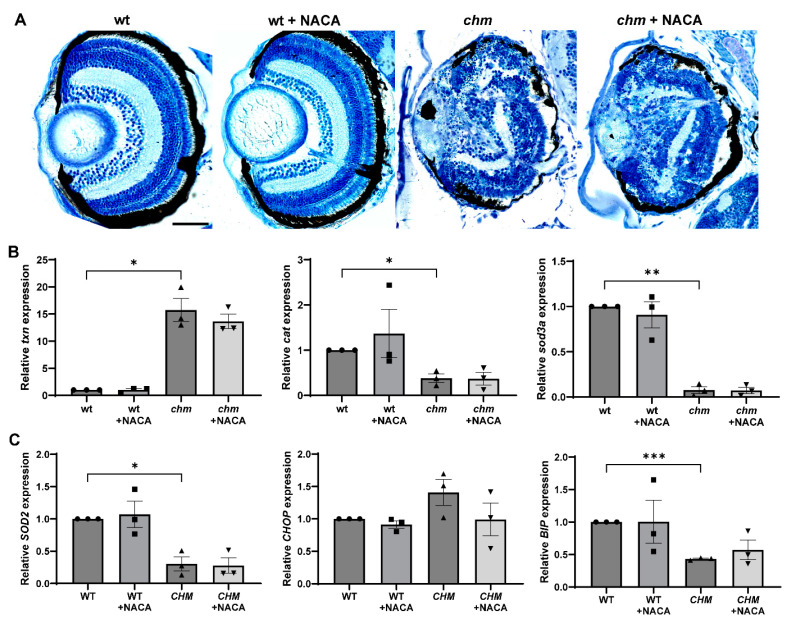
Analysis of oxidative stress in NACA-treated *chm^ru848^* fish and *CHM^Y42X^* cells. Fish were treated with 200 µg/mL of NACA at 10 hpf and collected at 5 dpf for (**A**) retinal histology and (**B**) RT-qPCR analysis of the oxidative stress markers *txn*, *cat* and *sod3a*. (**C**) Cells were treated with either 1 mM of NACA, and the expression of *SOD2* and the ER stress markers *CHOP* and *BIP* was analysed using RT-qPCR. Data are expressed as mean ± SEM from *n* = 3. * *p* ≤ 0.05, ** *p* ≤ 0.01, *** < *p* ≤ 0.001. Scale bar (50 μm), shown in wt corresponds to all images in panel.

N-acetylcysteine (NAC) is an antioxidant drug that was shown, in one study, to inhibit cone death by reducing oxidative damage in a mouse model of RP [20], and showed a slight improvement in cone function in a phase I clinical trial in RP patients [21]. NACA is an amide derivative of NAC with improved lipophilicity, antioxidant properties and an ability to cross the blood–brain barrier [22]. Fish were treated with 200 µg/mL of NACA at 10 h post fertilisation (hpf), and the retinal histology and expression of oxidative stress markers were assessed at 5 dpf. Treatment with NACA did not improve the retinal phenotype nor reduce oxidative stress in *chm^ru848^* fish (Figure 5A,B). Patient fibroblasts were treated with 1 mM of NACA for 24 h and the level of *SOD2* was analysed using RT-qPCR. NACA treatment did not increase the expression of *SOD2* in *CHM^Y42X^* cells (Figure 5C).

### 3.6. Levodopa (L-Dopa)

**Figure 6:**
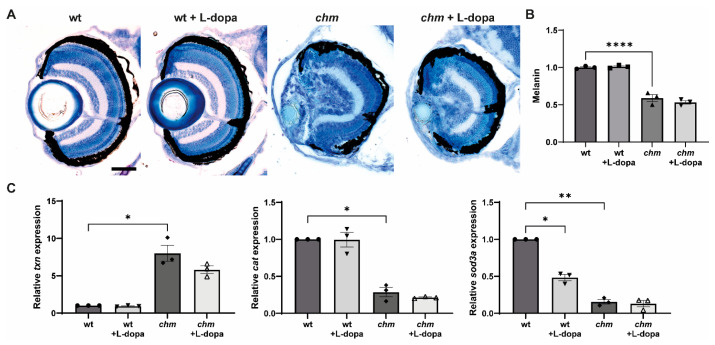
Analysis of melanin in L-dopa-treated *chm^ru848^* fish. Fish were treated with 1 mM of L-dopa at 3 dpf and collected at 5 dpf for (**A**) retinal histology, (**B**) total melanin quantification and (**C**) RT-qPCR analysis of the oxidative stress markers *txn*, *sod3a* and *cat*. Data are expressed as mean ± SEM from *n* = 3. * *p* ≤ 0.05, ** *p* ≤ 0.01, **** *p* ≤ 0.0001. Scale bar (50 μm), shown in wt corresponds to all images in panel.

L-dopa is a melanin precursor and has been shown to rescue retinal development, morphology and visual function in a murine model of oculocutaneous albinism (OCA) [23]. L-dopa activates the G-protein-coupled receptor 143 (GPR143), which is highly expressed in the RPE [24]. Fish were treated with 1 mM of L-dopa at 3 dpf and the total melanin levels were quantified in whole embryos at 5 dpf. The melanin levels in *chm^ru848^* fish were significantly reduced to 59 ± 3.9%, compared to wt fish (*p* < 0.0001); however, treatment with L-dopa did not cause any significant change in melanin levels (Figure 6B). Retinal histology was also assessed at 5 dpf; however, no improvements in the retinal phenotype were detected in the treated fish (Figure 6A). Melanin plays a key role in protecting the RPE from oxidative stress; therefore, the expression of oxidative stress markers was also analysed following L-dopa treatment; however, L-dopa did not reduce oxidative stress in *chm^ru848^* fish (Figure 6C). L-dopa was not tested in patient fibroblasts, due to a lack of the expression of GPR143 in dermal fibroblasts.

## 4. Discussion

In this study, we analysed the levels of oxidative stress in *chm^ru848^* zebrafish and found the significantly increased expression of *txn*, which encodes the thiol antioxidant thioredoxin. The reduced expression of genes encoding the antioxidant enzymes superoxide dismutase (sod3a) and catalase (cat) was also detected. Under conditions of mild stress, the expression and activity of antioxidant enzymes are increased to counteract the build-up of reactive oxygen species (ROS); however, when ROS levels become very high, expression is reduced, resulting in an inability to remove ROS and, ultimately, cell death [25]. This corresponds with previously reported increased levels of ROS and apoptotic cells in *chm^ru848^* zebrafish retinas [11]. In *CHM^Y42X^* patient fibroblasts, the SOD activity and expression of oxidative stress markers were largely comparable to WT cells; however, the expression of *SOD2* was significantly reduced. In a whole-metabolomic screening of 25 CHM patients, the levels of cysteine were reduced; however, there was an accumulation of hypotaurine, which is indicative of oxidative stress. In addition, the levels of antioxidants were also significantly reduced in CHM patient plasma [7].

The retina and RPE are exposed to high levels of oxidative stress as a result of daily light exposure and the phagocytosis of photoreceptor outer segments (POS). The maintenance of the RPE is reliant on a number of trafficking processes, including POS phagocytosis and melanosome transport. The silencing of the *CHM* gene in human foetal RPE cells resulted in the delayed clearance of POS [3], and impaired phagocytosis was also detected in the CHM-patient-derived-induced pluripotent stem cell (iPSC)-RPE [26] and *chm^ru848^* zebrafish [27]. Inefficient POS phagocytosis leads to an accumulation of undigested material and photooxidative lipofuscin, thereby increasing the levels of ROS in the cells. Rab27a, a target of REP1 (which accumulates in its unprenylated state in CHM patient lymphoblasts [28]), associates with myosin VIIa via the linker protein MyRIP to transport melanosomes to the RPE apical processes [29,30]. Fewer melanosomes were detected in the RPE apical processes of *Chm^Flox^, Tyr-Cre+* mice [6]. We also recently reported smaller melanosomes in the choroid of *Chm^null/WT^* mice, and significantly reduced melanosomes in the RPE and choroid of *chm^ru848^* zebrafish, along with reduced total melanin and a reduced expression of melanogenesis genes from 4 dpf [31]. Melanin, stored in melanosomes, absorbs light and protects the retina from photo-oxidative damage. Defects in melanosome transport, resulting in decreased melanin levels, can lead to a reduced ability to protect the RPE from oxidative stress. These defects in intracellular trafficking add to the already high level of free radicals in the RPE, resulting in increased oxidative stress and cellular damage.

In addition to oxidative stress, ER stress markers were also differentially expressed in *chm^ru848^* zebrafish and *CHM^Y42X^* fibroblasts. Binding immunoglobulin protein (BIP), also known as GRP78, is an ER chaperone and master regulator of the unfolded protein response (UPR) pathway. When unfolded proteins accumulate in the ER, causing ER stress, BIP is released from the three stress sensors, activating transcription factor 6 (ATF6), protein kinase RNA-like endoplasmic reticulum kinase (PERK), and inositol-requiring enzyme 1 (IRE1), which triggers a cascade of events leading to the inhibition of the translation and correct folding of proteins in order to reduce stress [32]. The expression of *bip*, *atf6* and *atf4* were all significantly increased in *chm^ru848^* zebrafish, indicating increased ER stress. Although increased BIP expression is typically associated with ER stress, reduced BIP expression, as detected in *CHM^Y42X^* fibroblasts, indicates a reduced ability to protect the cells from ER stress. In accordance, the expression of the downstream sensors *ATF6* and *ATF4* were not upregulated. If ER stress is prolonged and cellular homeostasis is not restored, apoptosis is induced via the activation of the C/EBP homologous protein (CHOP). Although cell death was not detected in *CHM^Y42X^* fibroblasts, *CHOP* expression was significantly increased, indicating the activation of the apoptosis pathway. Diminished BIP expression is also associated with ageing and neurodegenerative disorders [33]. Reduced BIP expression and increased CHOP protein expression were reported in the retina of aged WT mice [34]. Several Rab proteins are associated with ER, with various functions including Rab cycling, membrane tubulations and ER–Golgi trafficking [35]. A lack of REP1 can therefore lead to inefficient trafficking from the ER, resulting in an accumulation of proteins and the triggering of ER stress.

We screened a number of neuroprotectant drugs that have previously shown positive indications in other models of retinal disease for their ability to reduce ER and oxidative stress in both CHM models. The first was, TUDCA, which has been shown to preserve electroretinogram (ERG) b-waves and increase the outer nuclear layer thickness in *Rd10* and *Bbs1* mouse models of RP and Bardet–Biedl syndrome, respectively [14]. It has also been shown to slow down cone degeneration and reduce ER stress in *Lrat*^−/−^ mice [36]. TUDCA treatment in *chm^ru848^* fish reduced the expression of the ER stress markers *atf6* and *bip;* however, the expression levels were also reduced in fish treated with an equivalent volume of the vehicle DMSO. Although DMSO is commonly described as inducing ER stress, it is also known to act as a chemical chaperone and promote the folding of misfolded mutant proteins in the ER. Two percent DMSO was shown to restore the expression and promote folding of mutant E-cadherin [37], and increase the expression and activity of mutant RDH12 [38]. Another drug that has been shown to reduce ER stress is taurine, which reduced the starvation-induced expression of the ER stress markers *BIP/GRP78* and *CHOP* in ARPE-19 cells [19]. Taurine supplementation in a Royal College of Surgeons (RCS) rat model of RP also significantly increased the thickness of the outer nuclear layer and POS length, as well as reduced oxidative stress and the number of apoptotic cells [39]. However, taurine did not reduce the ER or oxidative stress in *CHM^Y42X^* fibroblasts or *chm^ru848^* fish in this study.

The antioxidant drug NACA was then tested for its ability to reduce oxidative stress in *chm^ru848^* fish and patient fibroblasts. NACA was shown to scavenge tert-Butyl hydroperoxide (tBHP)-induced ROS, reduce lipid peroxidation in ARPE-19 cells and slow photoreceptor degeneration in a *129/SvlmJ* light-induced mouse model of retinal degeneration [40]. NACA is currently being tested in a phase 1/2 clinical trial for patients with RP associated with Usher syndrome using Nacuity pharmaceuticals, under the name NPI-001 (NCT04355689). NACA did not improve the retinal histology or restore the expression of oxidative stress markers in either *chm^ru848^* zebrafish or *CHM^Y42X^* fibroblasts.

We also tested the melanin precursor L-dopa, which acts as an activator of GPR143, causing the increased expression of the pigment epithelium-derived factor in the RPE [24]. Oral L-dopa supplementation was shown to improve retinal function and morphology in OCA mice [23]; however, L-dopa supplementation did not improve the best corrected visual acuity (BCVA) in a clinical trial of 45 albinism patients [41]. The activation of GPR143 also triggers a reduction in the vascular endothelial growth factor (VEGF); it has therefore been proposed as a potential treatment for age-related macular degeneration (AMD) [42,43]. In a small proof-of-concept phase 2 clinical trial, following 6 months of L-dopa treatment, BCVA improved by 4.7 and 4.8 letters in two separate cohorts of patients with neovascular AMD (nAMD) [44]. The effect of L-dopa on melanin production and oxidative stress was investigated in *chm^ru848^* fish; however, there was no significant improvement in the retinal phenotype, nor an increase in the total melanin levels or a reduction in oxidative stress.

In conclusion, we have shown increased levels of oxidative and ER stress in two models of CHM, opening up avenues for alternative therapies targeting these pathways. However, some limitations to this study exist. While the *CHM^Y42X^* fibroblasts expressed signs of stress, the phenotype was relatively mild; therefore, investigations of other patient-derived fibroblasts would be beneficial to determine the levels of stress across multiple lines and whether this varies with mutation type. In addition, the levels of stress in fibroblasts may not be as high as those observed in the RPE and photoreceptors, due to greater compensation by REP2. A lack of the prenylation of the Rab proteins required in the retina may also cause a greater tissue-specific effect. Therefore, investigations into patient-derived iPSC-RPE and retinal organoids will provide a more relevant human model. Although the drugs tested here were not successful in reducing stress, *chm^ru848^* fish were proven to be a good model for drug screening due to the widespread severe phenotype. Zebrafish are amenable to high-throughput screening protocols due to their large breeding numbers, small size and rapid development of the retinal system, and have previously been used for drug screening in other models of retinal degeneration [45,46]. Zhang et al. carried out a large-scale phenotypic drug screen of 2934 neuroprotectants in a *rho:YFP-NTR*-tagged zebrafish model of RP, enabling the visualization of rod photoreceptor cell survival, and identified 113 hit compounds [45]. For future studies, using the fluorescent tagging of photoreceptors to monitor cell survival or the detection of ROS levels in the *chm^ru848^* retina will provide a more high-throughput readout measure to investigate the effectiveness of drugs. Any promising compounds identified in zebrafish can be escalated to mouse models; *Chm* knockout mice are embryonic lethal, but the heterozygous *Chm^null/WT^* female mouse model displays late-onset progressive retinal degeneration [47]. Finally, oxidative and ER stress are common features of multiple retinal dystrophies; therefore, any potential drugs identified in CHM screenings may be applicable to other forms of retinal degeneration.

## Figures and Tables

**Table 1:** RT-qPCR primer sequences.

Gene	Forward	Reverse
Human		
*ATF4*	TCAAACCTCATGGGTTCTCC	GTGTCATCCAACGTGGTCAG
*ATF6*	ACCCGTATTCTTCAGGGTGC	TCACTCCCTGAGTTCCTGCT
*CHOP*	GACCTGCAAGAGGTCCTGTC	TGTGACCTCTGCTGGTTCTG
*BIP*	GCCTGTATTTCTAGACCTGCC	TTCATCTTGCCAGCCAGTTG
*SOD1*	TAGCGAGTTATGGCGACGAAG	TGGTCCATTACTTTCCTTCTGCT
*SOD2*	GCTGGAAGCCATCAAACGTG	GCAGTGGAATAAGGCCTGTTG
*CAT*	CTCCGGAACAACAGCCTTCT	GAATGCCCGCACCTGAGTAA
*GAPDH*	ACAGTTGCCATGTAGACC	TTTTTGGTTGAGCACAGG
Zebrafish		
*atf4*	TGAGCACACTGAGGTTCCAG	GTCTTCACTCGGCCTTTGAG
*atf6*	TGATGAGGCACTGTCTCCAG	ATGGGTCTTTTTGCTGGTTG
*bip*	CAAGAAGAAGACGGGCAAAG	CTCCTCAAACTTGGCTCTGG
*txn*	GACCATCGGGCCGTACTTTA	CATAAAGCGGCCACATCCTGT
*cat*	ACGATGACAACGTGACCCAA	CCATCAGGTTTTGCACCATGC
*sod3a*	TCAAGTGCGTGCCATCCATA	CCGCCGGATAAGTCCTTGTT
*actin*	CGAGCTGTCTTCCCATCCA	TCACCAACGTAGCTGTCTTTCTG

## Data Availability

The data presented in this study are available in the article.
